# Action to Support Practices Implement Research Evidence (ASPIRE): protocol for a cluster-randomised evaluation of adaptable implementation packages targeting ‘high impact’ clinical practice recommendations in general practice

**DOI:** 10.1186/s13012-016-0387-5

**Published:** 2016-02-29

**Authors:** Thomas A. Willis, Suzanne Hartley, Liz Glidewell, Amanda J. Farrin, Rebecca Lawton, Rosemary R. C. McEachan, Emma Ingleson, Peter Heudtlass, Michelle Collinson, Susan Clamp, Cheryl Hunter, Vicky Ward, Claire Hulme, David Meads, Daniele Bregantini, Paul Carder, Robbie Foy, on behalf of the ASPIRE programme

**Affiliations:** 1 Leeds Institute of Health Sciences, University of Leeds, Leeds, LS2 9JT UK; 2 Leeds Institute of Clinical Trials Research, University of Leeds, Leeds, LS2 9JT UK; 3 School of Psychology, University of Leeds, Leeds, LS2 9LZ UK; 4 Bradford Institute for Health Research, Bradford Royal Infirmary, Bradford, BD9 6RJ UK; 5 West Yorkshire Research Service, Bradford Districts Clinical Commissioning Group, Douglas Mill, Bradford, BD5 7JR UK

**Keywords:** Primary care, Implementation, Cluster-randomised trial, Clinical guidelines, Diabetes, Prescribing, Atrial fibrillation

## Abstract

**Background:**

There are recognised gaps between evidence and practice in general practice, a setting which provides particular challenges for implementation. We earlier screened clinical guideline recommendations to derive a set of ‘high impact’ indicators based upon criteria including potential for significant patient benefit, scope for improved practice and amenability to measurement using routinely collected data. We aim to evaluate the effectiveness and cost-effectiveness of a multifaceted, adaptable intervention package to implement four targeted, high impact recommendations in general practice.

**Methods/design:**

The research programme Action to Support Practice Implement Research Evidence (ASPIRE) includes a pair of pragmatic cluster-randomised trials which use a balanced incomplete block design. Clusters are general practices in West Yorkshire, United Kingdom (UK), recruited using an ‘opt-out’ recruitment process. The intervention package adapted to each recommendation includes combinations of audit and feedback, educational outreach visits and computerised prompts with embedded behaviour change techniques selected on the basis of identified needs and barriers to change. In trial 1, practices are randomised to adapted interventions targeting either diabetes control or risky prescribing and those in trial 2 to adapted interventions targeting either blood pressure control in patients at risk of cardiovascular events or anticoagulation in atrial fibrillation. The respective primary endpoints comprise achievement of all recommended target levels of haemoglobin A1c (HbA1c), blood pressure and cholesterol in patients with type 2 diabetes, a composite indicator of risky prescribing, achievement of recommended blood pressure targets for specific patient groups and anticoagulation prescribing in patients with atrial fibrillation. We are also randomising practices to a fifth, non-intervention control group to further assess Hawthorne effects. Outcomes will be assessed using routinely collected data extracted 1 year after randomisation. Economic modelling will estimate intervention cost-effectiveness. A process evaluation involving eight non-trial practices will examine intervention delivery, mechanisms of action and unintended consequences.

**Discussion:**

ASPIRE will provide ‘real-world’ evidence about the effects, cost-effectiveness and delivery of adapted intervention packages targeting high impact recommendations. By implementing our adaptable intervention package across four distinct clinical topics, and using ‘opt-out’ recruitment, our findings will provide evidence of wider generalisability.

**Trial registration:**

ISRCTN91989345

**Electronic supplementary material:**

The online version of this article (doi:10.1186/s13012-016-0387-5) contains supplementary material, which is available to authorized users.

## Background

Clinical research can only benefit patient and population health if findings are incorporated into routine care. There are widely recognised failures to introduce effective new clinical practices quickly enough, consistently use those already proven to be effective or stop those found to be ineffective or even harmful. The gap between evidence and practice is an important problem for policy-makers, healthcare systems and research funders because it limits the health, social and economic impacts of clinical research [1]. Dissemination of best practice is necessary but seldom sufficient by itself to ensure implementation.

The context of general practice in the United Kingdom (UK) presents particular implementation challenges—given the limited practice organisational capacity, the increasing complexity of care and the dispersed and independent nature of practices. Many implementation studies focus on one condition (e.g. depression, back pain). This limits generalisability; it is uncertain how an intervention developed for one clinical condition will work for another [2, 3]. In 2012, we identified 107 clinical guidelines relevant to general practice produced by the National Institute for Health and Care Excellence (NICE) [4]. It is impracticable and inefficient to invent an implementation strategy for every new guideline. Implementation strategies are required which can be adapted to a range of targeted problems and sustainably integrated into available primary care systems and resources [5].

Action to Support Practices Implement Research Evidence (ASPIRE) is a research programme that aims to develop and evaluate an adaptable intervention package to target implementation of ‘high impact’ clinical practice recommendations in general practice. ASPIRE comprises five, sequential work packages:

1. Screening of NICE guidelines and associated quality standards to derive a set of ‘high impact’ indicators based on burden of illness, potential for significant patient benefit from improved practice, likelihood of cost savings without patient harm and feasibility of measuring change using routinely collected data [4].
2. Cross-sectional analysis of patient data to identify high impact recommendations with greatest scope for improvement (low adherence) and explore variations in adherence.
3. Interviews with primary care professionals to explore barriers to and enablers of adherence to selected high impact recommendations, matching of behaviour change techniques to identified barriers and enablers and development of an adaptable intervention package (based on audit and feedback, outreach educational visits and computerised prompts).
4. Evaluation of the effectiveness and cost-effectiveness of the adapted intervention package in targeting the implementation of high impact recommendations.
5. Conduct of a parallel process evaluation to examine intervention delivery, mechanisms of action and unintended consequences.

This protocol paper describes the randomised evaluation and summarises the process evaluation.

## Methods/design

### Study design and setting

**Fig. 1:**
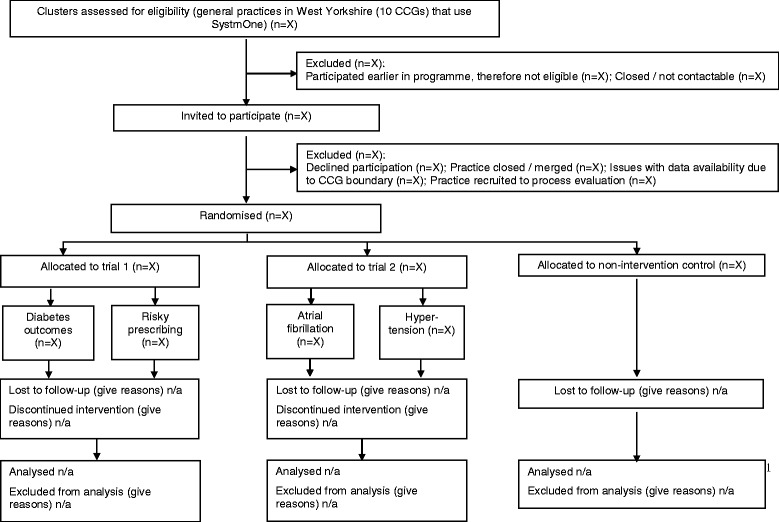
CONSORT 2010 checklist of information to include when reporting a cluster randomised trial

We are conducting a pair of pragmatic cluster-randomised trials with general practices as the unit of allocation. Cluster randomisation was chosen because the intervention is delivered at the practice level and aims to change clinical practice of the whole practice team. Balanced incomplete block designs equalise Hawthorne effects whilst maximising power and efficiency [6, 7] and also reduce the risk of overburdening practices through exposure to more than one intervention. In trial 1, practices will be randomised to adapted intervention packages targeting either diabetes control or risky prescribing. In trial 2, practices will be randomised to adapted intervention packages targeting either blood pressure control in patients at high risk of cardiovascular events or anticoagulation in atrial fibrillation. The balanced incomplete block design has two treatment arms per trial. Within both trials, all intervention packages were assumed to be independent in terms of their outcomes and each treatment arm will therefore be used as the control arm for the other treatment arm in the same trial (Fig. 1 and Additional file 1 detail a full CONSORT checklist). We are also including a fifth, non-intervention control group to further assess Hawthorne effects. Practices allocated to this arm will not receive any of the ASPIRE interventions, but we will examine outcomes via the same routinely collected data.

The study is set within West Yorkshire, which covers a socioeconomically and ethnically diverse population of approximately 2.2 million residents [8]. Around 330 general practices are organised within 10 clinical commissioning groups (CCGs), which have roles including the purchasing of specialist services and improving quality of primary care.

### Participants

General practices are eligible if they used the *SystmOne* computerised clinical system (TPP, http://www.tpp-uk.com/). Approximately two thirds of West Yorkshire practices use *SystmOne*. The use of a single system simplifies the process of data extraction and of implementing software support. We will exclude practices which had been involved in earlier stages of intervention development and piloting. We will also exclude from trial 2 practices in one CCG involved in a concurrent initiative addressing anticoagulation in atrial fibrillation.

### Recruitment and consent

Following consultations with our Programme Steering Group, our Public and Patient Involvement Panel and all 10 CCGs, we will use an ‘opt-out’ approach to recruit and consent general practices. Apart from facilitating the recruitment process, we judge that opt-out recruitment will enhance pragmatism and hence generalisability by resembling recruitment processes typically used by routine quality improvement initiatives [9]. We will send all eligible practices an invitation and information sheet via email. This outlines the purpose of the trial and what involvement entails and states that practices will be included unless they indicate otherwise. We will follow this with a duplicate pack (including a Freepost return envelope to return the opt-out notification) sent via recorded postal delivery. After 2 weeks, we will send reminders by both email and recorded postal delivery to all non-responding practices. Four weeks post-initial invitation, we will consider all practices which have not opted-out to have consented to participate. Patient-level consent is not required as all patient-level data are anonymised.

### Intervention

We broadly conceptualise the implementation intervention as comprising a range of behaviour change techniques (e.g. social comparison, information on health consequences, action planning). These will be embedded within a package based around three delivery mechanisms that possess a known evidence base and are increasingly central to routine implementation activities [7]: audit and feedback [7, 10], educational outreach visits [7, 11] and computerised prompts [7, 12]. We have further developed the packages iteratively through a series of exchanges with our professional and patient advisory groups and piloting in five general practices.

Following earlier work and consultations with professional and patient advisory groups, we decided to target the implementation of four high impact indicators:

- Control of HbA1c, blood pressure and cholesterol in type 2 diabetes [13, 14]
- Risky prescribing, largely focusing on avoiding adverse effects of non-steroidal anti-inflammatory drugs (NSAIDs) and anti-platelet drugs [15]
- Control of blood pressure in people at high risk of cardiovascular events [16] and
- Anticoagulation in people with atrial fibrillation [17].

We conducted semi-structured interviews with primary care professionals based on the Theoretical Domains Framework [18] to explore barriers and enablers to achieving the above indicators. For each indicator, we matched behaviour change techniques targeting clinicians to the most salient barriers and enablers and embedded them within intervention packages.

In brief, the audit and feedback component will comprise quarterly reports individualised for each practice and presenting their performance against targeted clinical indicators, together with motivational messages based on key theoretical domains identified by our qualitative research. The educational outreach visits will be delivered by trained pharmacist facilitators and involve discussion of practice performance and development of action plans. The computerised support will include modified versions of the searches used to prepare the audit reports and, for risky prescribing only, automated messages triggered by potentially risky prescribing combinations. We will also offer practices up to 2 days of additional pharmacist support. The nature of this support will be decided in discussion with practice but can include review of patient notes or modification of computerised searches to identify specific patient groups to invite for consultation. All practices in the intervention arms will be sent electronic and postal copies of the feedback reports. They will also be offered educational outreach, additional pharmacist support, searches to identify patients and, where applicable, computerised prompts. Given the pragmatic nature of these trials, the acceptance and engagement with all components is optional. We will further describe intervention development and content in a separate paper.

### Outcomes

The primary endpoints are as follows:

- The proportion of patients with type 2 diabetes achieving all three of the following treatment targets: blood pressure below 140/80 mmHg (or 130/80 mmHg if there is kidney, eye or cerebrovascular damage), HbA1c value below or equal to 59 mmol/mol and cholesterol level below or equal to 5.0 mmol/l (trial 1)
- A composite measure of nine indicators of high-risk NSAID and anti-platelet prescribing: prescribing a traditional oral NSAID or low-dose aspirin in patients with a history of peptic ulceration without co-prescription of gastro-protection; prescribing a traditional oral NSAID in patients aged 75 years or over without co-prescription of gastro-protection; prescribing of a traditional oral NSAID and aspirin in patients aged 65 years or over without co-prescription of gastro-protection; prescribing of aspirin and clopidogrel in patients aged 65 years or over without co-prescription of gastro-protection; prescribing of warfarin and a traditional oral NSAID; prescribing of warfarin and low-dose aspirin or clopidogrel without co-prescription of gastro-protection; prescribing an oral NSAID in patients with heart failure; prescribing an oral NSAID in patients prescribed both a diuretic and an angiotensin-converting-enzyme inhibitor (ACE-I) or angiotensin receptor blocker (ARB) and prescribing an oral NSAID in patients with chronic kidney disease (CKD; trial 1)
- The proportion of patients with satisfactorily controlled blood pressure according to recommended targets: under 140/90 mmHg in patients aged under 80 years with hypertension; under 150/90 mmHg in patients aged 80 years and over with hypertension; under 140/80 mmHg in patients aged under 80 years with diabetes, under 130/80 mmHg if there are complications of diabetes; under 130/80 mmHg in patients aged under 80 years with chronic kidney disease and proteinuria; under 140/90 mmHg in patients aged under 80 years with coronary heart disease; under 140/90 mmHg in patients aged under 80 years with peripheral arterial disease; under 140/90 mmHg in patients aged under 80 years with a history of stroke/transient ischemic attack and under 140/90 mmHg in patients aged under 80 years with a cardiovascular disease risk of 20 % or higher (trial 2) and
- A composite of the proportion of men with atrial fibrillation and a CHA_2_DS_2_-VASc score of 1 prescribed anticoagulation therapy and the proportion of all people with atrial fibrillation and a CHA_2_DS_2_-VASc score of 2 or above prescribed anticoagulation therapy (trial 2).

Secondary endpoints comprise the following:

- The effects on separate individual indicators that contribute to composite outcomes (e.g. individual blood pressure, HbA1c and cholesterol targets in patients with diabetes)
- The effects on recorded processes of care (the proportion of patients with type 2 diabetes achieving all nine of the following recommended processes of care in the previous 12 months: blood pressure recording, HbA1c recording, total cholesterol recording, urine albumin to creatinine ratio (ACR) or protein to creatinine ratio (PCR) or proteinuria coding, estimated glomerular filtration rate (eGFR) or serum creatinine testing, foot care review, retinal screening, body mass index recording, smoking status)
- The effects on continuous intermediate clinical outcomes (i.e. value of last recorded blood pressure, HbA1c, cholesterol)

In addition, we will gather selected data from the Quality and Outcomes Framework (QOF; 2015–2016 indicator list (Table 1). The QOF is a performance management system whereby general practices are remunerated according to achievement of targets reflecting quality of care across four domains of clinical, organisational, patient experience and additional services [19]. Practice data collection for QOF operates on an annual cyclical basis from 1 April to 31 March. Several indicators map onto our trial recommendations (Table 1). We have also selected a series of non-trial-related indicators that represent different aspects of care and service delivery. These will be used to measure unintended impacts on quality of care and relate to four areas: coronary heart disease (an example of a long-term physical condition), mental health (long-term non-physical condition), smoking (health promotion) and asthma (long-term physical condition). All outcomes will be assessed at 1 year following randomisation.

**Table 1:** List of indicators from the Quality and Outcomes Framework 2015–2016 included in the ASPIRE analysis

Domain	QOF indicator number (2015–2016)	Indicator
Atrial fibrillation	AF006	The percentage of patients with atrial fibrillation in whom stroke risk has been assessed using the CHA2DS2-VASc score risk stratification scoring system in the preceding 12 months (excluding those patients with a previous CHADS2 or CHA2DS2-VASc score of 2 or more).
Atrial fibrillation	AF007	In those patients with atrial fibrillation with a record of a CHA2DS2-VASc score of 2 or more, the percentage of patients who are currently treated with anticoagulation drug therapy.
Secondary prevention of coronary heart disease	CHD002	The percentage of patients with coronary heart disease in whom the last blood pressure reading (measured in the preceding 12 months) is 150/90 mmHg or less.
Secondary prevention of coronary heart disease	CHD005	The percentage of patients with coronary heart disease with a record in the preceding 12 months that aspirin, an alternative anti-platelet therapy or an anti-coagulant is being taken.
Secondary prevention of coronary heart disease	CHD007	The percentage of patients with coronary heart disease who have had influenza immunisation in the preceding 1 August to 31 March.
Hypertension	HYP006	The percentage of patients with hypertension in whom the last blood pressure reading (measured in the preceding 12 months) is 150/90 mmHg or less.
Stroke and transient ischemic attack	STIA003	The percentage of patients with a history of stroke or TIA in whom the last blood pressure reading (measured in the preceding 12 months) is 150/90 mmHg or less.
Diabetes mellitus	DM002	The percentage of patients with diabetes, on the register, in whom the last blood pressure reading (measured in the preceding 12 months) is 150/90 mmHg or less.
Diabetes mellitus	DM003	The percentage of patients with diabetes, on the register, in whom the last blood pressure reading (measured in the preceding 12 months) is 140/80 mmHg or less.
Diabetes mellitus	DM004	The percentage of patients with diabetes, on the register, whose last measured total cholesterol (measured within the preceding 12 months) is 5 mmol/l or less.
Diabetes mellitus	DM006	The percentage of patients with diabetes, on the register, with a diagnosis of nephropathy (clinical proteinuria) or micro-albuminuria who are currently treated with ACE-I (or ARBs).
Diabetes mellitus	DM007	The percentage of patients with diabetes, on the register, in whom the last IFCC-HbA1c is 59 mmol/mol or less in the preceding 12 months.
Diabetes mellitus	DM008	The percentage of patients with diabetes, on the register, in whom the last IFCC-HbA1c is 64 mmol/mol or less in the preceding 12 months.
Diabetes mellitus	DM009	The percentage of patients with diabetes, on the register, in whom the last IFCC-HbA1c is 75 mmol/mol or less in the preceding 12 months.
Diabetes mellitus	DM0012	The percentage of patients with diabetes, on the register, with a record of a foot examination and risk classification: (1) low risk (normal sensation, palpable pulses), (2) increased risk (neuropathy or absent pulses), (3) high risk (neuropathy or absent pulses plus deformity or skin changes in previous ulcer) or (4) ulcerated foot within the preceding 12 months.
Diabetes mellitus	DM0014	The percentage of patients newly diagnosed with diabetes, on the register, in preceding 1 April to 31 March who have a record of being referred to a structured education programme within 9 months after entry on to the diabetes register
Diabetes mellitus	DM0018	The percentage of patients with diabetes, on the register, who have had influenza immunisation in the preceding 1 August to 31 March.
Mental health	MH002	The percentage of patients with schizophrenia, bipolar affective disorder and other psychoses who have a comprehensive care plan documented in the record, in the preceding 12 months, agreed between individuals, their family and/or carers as appropriate.
Mental health	MH003	The percentage of patients with schizophrenia, bipolar affective disorder and other psychoses who have a record of blood pressure in the preceding 12 months.
Smoking	SMOK002	The percentage of patients with any or any combination of the following conditions: coronary heart disease (CHD), peripheral arterial disease (PAD), stroke or transient ischemic attack (TIA), hypertension, diabetes, chronic obstructive pulmonary disease (COPD), chronic kidney disease (CKD), asthma, schizophrenia, bipolar affective disorder or other psychoses whose notes record smoking status in the preceding 12 months.
Smoking	SMOK004	The percentage of patients aged 15 or over who are recorded as current smokers who have a record of an offer or support and treatment within the preceding 24 months.
Smoking	SMOK005	The percentage of patients with any or any combination of the following conditions: CHD, PAD, stroke or TIA, hypertension, diabetes, COPD, CKD, asthma, schizophrenia, bipolar affective disorder or other psychoses who are recorded as current smokers who have a record of an offer of support and treatment within the preceding 12 months
Asthma	AST003	The percentage of patients with asthma, on the register, who have had an asthma review in the preceding 12 months that includes an assessment of asthma control using the three Royal College of Physician questions.

### Data collection

#### Primary and secondary endpoints

We will use anonymised patient health records, extracted remotely from participating practices to obtain data on indicator adherence. The data will be collected via the data quality team at the National Health Service (NHS) Yorkshire & Humber Commissioning Support Unit. Data extraction and collation procedures are performed under the appropriate Information Governance Guidance, in line with Caldicott Principles.

Data queries are developed by mapping inclusion and exclusion criteria to the numerators and denominators for each indicator and building the search within *SystmOne*. Reports are generated by a data analyst to define output for each indicator numerator and denominator and extracted in a standardised format. The outputs are anonymised and checked for accuracy prior to transfer to the Leeds Institute of Clinical Trials Research (LICTR) via a secure file transfer system.

Pre-intervention data will be extracted for randomisation stratification and then continue quarterly to inform the content of feedback reports. The final extraction will be used for endpoint analysis.

#### Practice and patient characteristics

Data on practice characteristics will be obtained from publicly available sources (http://www.hscic.gov.uk) and include practice list size (number of registered patients), number of general practitioner (GP) partners, number of salaried GPs, practice teaching status, practice level Index of Multiple Deprivation (IMD), ethnic profile of practice register, achievement of QOF indicators, patient satisfaction (proportion who would recommend practice to others), patient-rated practice accessibility (proportion able to speak with GP or nurse within 48 hours of approach) and practice prescribing costs. Patient characteristics will be extracted alongside adherence data and include age, sex, co-morbidity (number of QOF disease registers patient is included in) and polypharmacy (number of repeat prescriptions).

#### Intervention fidelity

Intervention delivery and fidelity will be monitored throughout the trials. Structured logs record the number of outreach sessions delivered, number of staff attending and whether feedback reports were (i) received and (ii) used, as well as any additional support requested by the practice. Outreach facilitators will complete standard review forms following each visit. Reasons for declining an outreach visit will also be documented. We will collect data on which practices accept the invitation to join indicator-specific *SystmOne* organisational groups. Acceptance is required in order to access the searches, as well as any available computer protocols. These lists will be monitored during the trials and reminders sent to practices which have not joined.

### Sample size

Data collected for the second ASPIRE work package (cross-sectional assessment in a random sample of practices of adherence to selected clinical indicators) was used to estimate mean cluster size (number of targeted patients per practice by indicator), coefficient of variation, intra-cluster correlation coefficient and control group achievement rates (Table 2). Depending on the recommendation, assumed intra-cluster correlation coefficients (ICC) ranged from 0.03 to 0.06, mean cluster sizes from 55 to 800 and the coefficient of variation of cluster sizes from 0.6 to 0.79 (Table 2). The median effect sizes on processes and outcomes of care for a range of single interventions in guideline implementation studies are around 4–9 % [20, 21]. Given the enhancements we are making to the intervention packages and that we are targeting recommendations with greater scope for improvement [10], we judge that an estimated effect size of 15 % for outcomes related to diabetes, hypertension and atrial fibrillation are realistic and clinically relevant. Control group adherence rates in risky prescribing are considerably higher (Table 2), and considering a potential ceiling effect, we estimated 5 % to be a realistic and clinically relevant effect size for this intervention.

**Table 2:** Key sample size assumptions

	Atrial fibrillation	Blood pressure control	Risky prescribing	Diabetes control
Mean number of patients per practice (cluster size)	55	800	420	280
Coefficient of variation (CV) of cluster size	0.79	0.67	0.65	0.6
Intra-cluster correlation coefficient (ICC)	0.06	0.06	0.03	0.06
Control group adherence	0.6	0.72	0.89	0.43

In order to achieve 90 % power, and allowing for an alpha error rate of 2.5 % (to adjust for two outcome comparisons in each trial) and a 10 % drop-out rate, we require 40 clusters per arm in trial 1 (diabetes and risky prescribing) and 32 clusters per arm in trial 2 (hypertension and atrial fibrillation). We therefore aim to recruit 144 practices (Table 3). We allowed for the possibility of a fifth arm because we anticipated achieving above-target recruitment levels using the opt-out approach.

**Table 3:** Trial design and number of practices required

Trial 1		Trial 2		Non-intervention control
Diabetes control	Risky prescribing	Blood pressure control	Anticoagulation in atrial fibrillation	
40	40	32	32	[Number dependent on recruitment beyond that required for intervention sample]

### Randomisation

Randomisation will be conducted using a computer-generated minimisation programme (incorporating a random element) to ensure arms are balanced. It will follow a two-stage process. First, practices will be stratified by CCG and practice list size (defined as above/below the West Yorkshire median list size of 6562 patients) and then randomised to trial 1, trial 2 or the no-intervention arm. Second, practices in trials 1 and 2 will then be stratified again by CCG, practice list size and pre-intervention adherence to the two relevant targeted clinical areas. They will then be randomised to the individual arms within each trial. Cluster randomisation will be performed at LICTR by the trial statistician.

For both trials, each practice will act as a control practice for the other corresponding arm in the trial to which they are randomised. Practices in the no-intervention arm will operate as additional controls.

General practice staff and trial personnel involved in the delivery of the intervention will, of necessity, be aware of assignment to allocation but collection of outcomes for the primary endpoints will be blind.

### Statistical analysis

All analyses and data summaries will be conducted on the intention-to-treat (ITT) population, defined as all randomised practices regardless of compliance with the intervention or withdrawal from the study. No formal interim analyses are planned, and final analysis will take place when all available data are received from the final extract.

#### Primary analysis

As the trials are cluster randomised, the primary outcome measures (levels of adherence to the four indicators) will be compared between the intervention and control groups using two-level binary logistic models, with patients nested within general practices. Effect sizes and 95 % confidence intervals will be reported.

#### Secondary analyses

Secondary analyses will be undertaken using both binary and continuous outcome data (e.g. blood pressure levels). For binary outcome data, we will also be using two-level binary logistic models, for continuous data, two-level linear models. Effect sizes and 95 % confidence intervals will be reported.

### Cost-effectiveness analysis

We plan to conduct economic analyses for three of the four intervention packages: diabetes control, risky prescribing and blood pressure control. We had to limit the number of outcomes modelled because of our limited resources and selected three based on availability of existing models.

Economic modelling has the primary objective of identifying the interventions’ incremental cost-effectiveness ratios. In order to assess the impact of the intervention over a lifetime horizon, longer-term models will be developed for each of the three selected areas. The decision analytic models will be informed by published evidence on costs (taking a UK health and social care perspective as per NICE guidelines) and utility values (used to construct quality-adjusted life years [QALYs]). Values generated for time periods greater than 12 months will be discounted at the 3.5 % rate.

The economic evaluations will make use of an adherence parameter informed by effectiveness data obtained from the study. The parameter will reflect the impact of the intervention; for example, an improvement in adherence should lead to an improvement in outcomes (QALYs) predicted by the model.

The value of the intervention will depend upon the value of the behaviour it promotes. Thus, its cost-effectiveness will be application-specific, just as drugs which treat multiple conditions do not have a single cost-effectiveness. The main outcome of interest will be cost per QALY and net monetary benefit. We will conduct extensive deterministic sensitivity analysis and probabilistic sensitivity analysis to assess uncertainty surrounding estimates of cost-effectiveness.

### Trial and data monitoring

Trial supervision includes a core project team, a Trial Management Group (TMG; consisting of a subset of the project team and relevant independent experts in statistics and health economics) and an independent Trial Steering Committee (TSC). For a trial of this nature and duration, a separate Data Monitoring and Ethics Committee is not required. Rather, the TSC will adopt a safety monitoring role, with the constitution of a sub-committee to review safety issues where this becomes necessary. Any clinical governance issues pertaining to aspects of routine management will be brought to the attention of the TSC and, where applicable, to individual practices.

### Process evaluation

We will conduct a process evaluation alongside the trials to:

- Describe how the intervention packages are implemented and the extent to which they become embedded in routine systems and practice
- Explore whether the interventions worked as predicted or by an alternative means and
- Identify unintended consequences of the delivery and implementation of the intervention packages in practice.

#### Recruitment and consent

We will recruit a subset of general practices not involved in the trials. Email invitations will be followed up by telephone calls and practice-level informed consent obtained. Practices will be reimbursed for their time and commitment over the study period.

We aim to recruit eight practices, providing a sample of two practices per clinical topic. We will purposively select practices to ensure a spread across CCGs and a range of practice sizes (patient list size and number of practice staff). Where individual practice staff members participate in audio-recorded interviews, individual written consent will be obtained.

#### Method

Process evaluation practices will be randomised by the trial statistician to receive an intervention package targeting one of the four indicators. These will be delivered in identical fashion to, and in parallel with, the trial practices.

In preparation for data collection at the process evaluation practices, we will develop a logic model to capture how we expect the intervention package and its constituent components to be received and how it may bring about change [22, 23].

Throughout the intervention period, we will conduct in-depth work with process evaluation practices to observe and monitor how the interventions are received and implemented. Data collection will proceed via observations, interviews and the collection of administrative data [24].

- Observations: where possible, settings where practice staff discuss or interact with the intervention package and/or clinical topic will be observed (e.g. educational outreach sessions, practice meetings), and detailed field notes generated to capture how staff receive and interact with the intervention package at a group level [25]. No individual patient consultations or data will be collected.
- Interviews: staff will be interviewed individually about their role in the practice, their interaction with the intervention, whether it is perceived to influence behaviour in relation to the clinical topic, if and how the intervention has been incorporated into routine practice, and any unintended consequences arising from the intervention. Interviews will be both semi-structured and open-ended to help capture intended and unintended mechanisms. A range of practice staff will be interviewed in each participating practice (up to 12 individuals per site), including staff with administrative, clinical and managerial roles. Interviews will take place at three time points, twice to coincide with delivery of key intervention components (educational outreach and feedback report delivery) and, finally, after trial end. Interviews will be audio-recorded and transcribed verbatim.
- Administrative data: information relating to the intervention package and/or target clinical recommendations will be collected. Examples include meeting minutes, practice protocols around a clinical topic and communications (e.g. emails) about the clinical topic or intervention package.

#### Analysis

Data will be collated to produce chronological reconstructions of the process and outcomes of receiving and implementing the intervention package. This will be on a case by case basis, wherein each practice acts as a case study [26]. Data will be analysed using a framework approach [27] based on our pre-specified theories of how the intervention package will work (outlined in the logic model) and drawing on normalisation process theory to understand the process of implementation in each practice [28]. We also aim to identify and explore any unintended consequences and mechanisms of the intervention package during analysis.

### Ethical review

Ethical approval has been obtained through the UK National Research Ethics Service (14/SC/1393) (Additional file 2). NHS Research and Development approvals were granted by the NHS Yorkshire & Humber Commissioning Support Unit on behalf of all the CCGs in West Yorkshire (approved 30 January 2015).

## Trial status

The trials are currently in progress. No endpoint data collection nor cleaning has yet occurred.

## Discussion

The ASPIRE trials will provide robust evidence about the effectiveness and cost-effectiveness of an implementation package for primary care targeting ‘high impact’ indicators, as well as the further insights generated by the process evaluation.

## Additional files

Additional file 1: CONSORT 2010 checklist of information to include when reporting a cluster randomised trial. (DOCX 28 kb)

Additional file 2: Confirmation of ethical approval. (PDF 262 kb)

## Abbreviations

ACE-I: angiotensin-converting-enzyme inhibitor
ACR: albumin to creatinine ratio
ARB: angiotensin receptor blocker
ASPIRE: Action to Support Practices Implementing Research Evidence
CCG: clinical commissioning group
CHD: coronary heart disease
CKD: chronic kidney disease
CONSORT: Consolidated Standards of Reporting Trials
COPD: chronic obstructive pulmonary disease
CRN: Clinical Research Network
eGFR: estimated glomerular filtration rate
GP: general practitioner
ICC: intra-cluster correlation coefficient
IFCC-HbA1c: International Federation of Clinical Chemistry and Laboratory Medicine-Glycated Haemoglobin
IMD: index of multiple deprivation
ITT: intention-to-treat
LICTR: Leeds Institute of Clinical Trials Research
NHS: National Health Service
NICE: National Institute for Health and Care Excellence
NIHR: National Institute for Health Research
NRES: National Research Ethics Service
NSAID: non-steroidal anti-inflammatory drug
PAD: peripheral arterial disease
PCR: protein to creatinine ratio
QALYs: quality-adjusted life years
QOF: Quality and Outcomes Framework
REC: Research Ethics Committee
TIA: transient ischemic attack
TMG: Trial Management Group
TSC: Trial Steering Committee
UK: United Kingdom
