# Supplementary material for: Action to Support Practices Implement Research Evidence (ASPIRE): protocol for a cluster-randomised evaluation of adaptable implementation packages targeting ‘high impact’ clinical practice recommendations in general practice
Source: Implement Sci. 2016 Feb 29;11:25. doi: 10.1186/s13012-016-0387-5 (PMC4770678; doi:10.1186/s13012-016-0387-5)
Supplement: Supplementary file 2 — Confirmation of ethical approval. (PDF 262 kb) [file 13012_2016_387_MOESM2_ESM.pdf]

NHS
Health Research Authority
NRES Committee Yorkshire & The Humber - Bradford Leeds
Jarrow REC Centre
Jarrow Business Centre
Room 002
Rolling Mill Road
Jarrow
NE32 3DT

Telephone: 0191 428 3565

1 December 2014

Professor Robbie Foy
Professor of Primary Care
c/o Clinical Trials Research Unit
Leeds Institute of Clinical Trials Research
University of Leeds, Leeds
LS2 9JT

Dear Professor Foy

**Study title:**	**A cluster-randomised evaluation of adaptable implementation packages targeting high impact clinical practice recommendations in general practice**
**REC reference:**	**14/YH/1265**
**Protocol number:**	**TBC**
**IRAS project ID:**	**167031**

The Research Ethics Committee reviewed the above application at the meeting held on 18 November 2014. Thank you for attending to discuss the application with key investigator Dr Thomas Willis.

We plan to publish your research summary wording for the above study on the HRA website, together with your contact details, unless you expressly withhold permission to do so. Publication will be no earlier than three months from the date of this favourable opinion letter. Should you wish to provide a substitute contact point, require further information, or wish to make a request to postpone publication, please contact the REC Manager Ms Gillian Mayer, nrescommittee.yorkandhumber-bradfordleeds@nhs.net.

Ethical opinion

The members of the Committee present gave a **Favourable** ethical opinion of the above research on the basis described in the application form, protocol and supporting documentation, subject to the conditions specified below. .

Conditions of the favourable opinion

The favourable opinion is subject to the following conditions being met prior to the start of the study.

Management permission or approval must be obtained from each host organisation prior to the start of the study at the site concerned.

Management permission ("R&D approval") should be sought from all NHS organisations involved in the study in accordance with NHS research governance arrangements.

Guidance on applying for NHS permission for research is available in the Integrated Research Application System or at http://www.rdforum.nhs.uk.

Where a NHS organisation's role in the study is limited to identifying and referring potential participants to research sites ("participant identification centre"), guidance should be sought from the R&D office on the information it requires to give permission for this activity.

For non-NHS sites, site management permission should be obtained in accordance with the procedures of the relevant host organisation.

Sponsors are not required to notify the Committee of approvals from host organisations.

Registration of Clinical Trials

All clinical trials (defined as the first four categories on question 2 of the IRAS filter page) must be registered on a publically accessible database within 6 weeks of recruitment of the first participant (for medical device studies, within the timeline determined by the current registration and publication trees).

There is no requirement to separately notify the REC but you should do so at the earliest opportunity e.g. when submitting an amendment. We will audit the registration details as part of the annual progress reporting process.

To ensure transparency in research, we strongly recommend that all research is registered but for non-clinical trials this is not currently mandatory.

If a sponsor wishes to contest the need for registration they should contact Catherine Blewett (catherineblewett@nhs.net), the HRA does not, however, expect exceptions to be made. Guidance on where to register is provided within IRAS.

Ethical review of research sites

NHS Sites

The favourable opinion applies to all NHS sites taking part in the study taking part in the study, subject to management permission being obtained from the NHS/HSC R&D office prior to the start of the study (see "Conditions of the favourable opinion" below).

Summary of discussion at the meeting

The Committee considered this application covered an interesting area to study.

Social or scientific value; scientific design and conduct of the study

The Committee noted this application is part of an ongoing project and requested a general update of progress to date including timelines.

You informed that you had completed part of this five year programme which aims to promote understanding and change professional practice in line with current national guidelines. The aim is to undertake pragmatic research into effectiveness of clinical interventions and determine what is typical in GP surgeries and increase standardisation of treatment. Although it is highly unlikely that professional misconduct may be identified, you stated this work needs to be undertaken. The study includes five sequences and the first part involved a review of all NICE guidelines and factors relevant to primary care were extracted and then a few were chosen which you considered may make a difference. The second phase randomly sampled practices in West Yorkshire and patient records were used to measure performance and review the data to identify areas which could be improved. Eight of those practices were then chosen to progress to the next phase. You

confirmed that the programme involved review of four interventions - hypertension, atrial fibrillation, diabetes and 'risky prescribing', which all have different elements, as further evidence is required on these areas.

The Committee queried if both treatment arms in the trial would receive the same package.

You confirmed this and noted that there would be a different topic to focus on.

The Committee asked how you would assess if this work has the potential to make a difference or not.

You stated that you would assess quality outcomes of the existing data and noted that no new data will be collected from patients.

The Committee queried what the term 'economic evaluation' referred to.

You explained that you have data from this project feeding into economic evaluation to determine if it is worth the money and determine the level of change accomplished. You confirmed this would be based on the cost of the intervention.

The Committee asked if you would estimate cost savings on the results obtained.

You confirmed that you would not and you would be using aggregate data to value and estimate the quality of life.

It was queried if the evidence indicated that the number of stokes per year has reduced.

You confirmed that some data had shown a five to ten percent improvement in the number of strokes recorded, however you considered that this could be improved further.

Recruitment arrangements and access to health information, and fair participant selection

The Committee noted that study information will be sent to potential participants by recorded delivery to ensure the package has been received and they are given thirty days to consider involvement. It was queried if the targeted individuals would be given the chance to opt-out of the study

You confirmed that participants will be randomised and can withdraw from the study at any time. You clarified that the opt-out approach had been included in an earlier phase of the study and anonymised data was collected.

It was noted that a previous application for this programme had been reviewed and approved.

Approved documents

The documents reviewed and approved at the meeting were:

Document	*Version*	*Date*
Covering letter on headed paper [ASPIRE ethics application covering letter_141105_signed]		06 November 2014
Evidence of Sponsor insurance or indemnity (non NHS Sponsors only) [UoL_Indeminity_Clinical Trials Confirmation_2014_2015]		17 September 2014
Letter from funder [PGfAR - Standard Contract Cover Letter RP-PG-1209-10040]		05 March 2012
Letter from sponsor [Sponsor Peer Review Letter_141103]		03 November 2014
Letters of invitation to participant [ASPIRE Invitation Letter v1.0 031114]	1.0	03 November 2014

Letters of invitation to participant [ASPIRE Reminder Letter v1.0 031114]	1.0	03 November 2014
Other [Email of Support From PPI Chair]		29 October 2014
Other [YHCS Letter of Support]		24 October 2014
Other [Stage 2 Notification Letter RP-PG-1209-10040 Foy]		29 July 2011
Other [RP-PG-1209-10040_Feedback Report]		
Other [RP-PG-1209-10040 HE evaluation]		
Other [NIHR programme primary care reviews]		
Other [Response to panel Foy RP-PG-1209-10040]		06 September 2011
Other [Response to panel health economics Foy RP-PG-1209-10040]		
Participant consent form [ASPIRE Opt Out Form v1.0 031114]	1.0	03 November 2014
Participant information sheet (PIS) [ASPIRE_Participant information sheet - practice_v1.0_03 11 14]	1.0	03 November 2014
REC Application Form [REC_Form_06112014]		06 November 2014
Research protocol or project proposal [ASPIRE_protocol_v2.0_14 11 05]	2.0	05 November 2014
Summary CV for Chief Investigator (CI) [Robbie Foy 2-page CV 140827 signed]		27 August 2014

Membership of the Committee

The members of the Ethics Committee who were present at the meeting are listed on the attached sheet.

After ethical review

Reporting requirements

The attached document "After ethical review – guidance for researchers" gives detailed guidance on reporting requirements for studies with a favourable opinion, including:

- Notifying substantial amendments
- Adding new sites and investigators
- Notification of serious breaches of the protocol
- Progress and safety reports
- Notifying the end of the study

The HRA website also provides guidance on these topics, which is updated in the light of changes in reporting requirements or procedures.

User Feedback

The Health Research Authority is continually striving to provide a high quality service to all applicants and sponsors. You are invited to give your view of the service you have received and the application procedure. If you wish to make your views known please use the feedback form available on the HRA website: http://www.hra.nhs.uk/about-the-hra/governance/quality-assurance/

HRA Training

We are pleased to welcome researchers and R&D staff at our training days – see details at http://www.hra.nhs.uk/hra-training/

**14/YH/1265**	**Please quote this number on all correspondence**

With the Committee's best wishes for the success of this project.

Yours sincerely
pp

Dr Janet Holt
Chair

E-mail: nrescommittee.yorkandhumber-bradfordleeds@nhs.net

Enclosures: *List of names and professions of members who were present at the meeting and those who submitted written comments*

'After ethical review – guidance for researchers'

Copy to: *Mr Paul Carder - NHS West and South Yorkshire and Bassetlaw Commissioning Support Unit*

Ms Clare Skinner – R&D Dept, Leeds University

NRES Committee Yorkshire & The Humber - Bradford Leeds

Attendance at Committee meeting on 18 November 2014

Committee Members:

Name	*Profession*	*Present*	*Notes*
Mr Sharif Al-Ghazal	Consultant Plastic Surgeon	No	
Professor Diana Anderson	Professor of Biomedical Sciences	No	
Ms Ruby Bhatti	Consultant Solicitor	No	
Ms Anne Davies (Vice Chair)	Clinical Research Consultant	No	
Dr David Dawson	Consultant in Anaesthetics	No	
Dr Stan Dobrzanski	Clinical Services Manager Pharmacy	Yes	
Mrs Jenny Foggin	Senior Governance & Corporate Affairs Officer	Yes	
Ms Rebecca Forster	Research Fellow	Yes	
Mr Simon Gelsthorpe	Head of Psychological Therapies	Yes	
Mr Jay Gokhale	Consultant General Surgeon	Yes	
Dr Janet Holt (Chair)	Senior Lecturer	Yes	
Dr Kirste Mellish	Research Programme Manager	No	
Mr Andrew Scally	Senior Lecturer	Yes	
Dr Christopher Skidmore (Alternate Vice Chair)	Retired University Lecturer	Yes	
Mr Robert Thompson	Lay Member	No	

Also in attendance:

Name	*Position (or reason for attending)*
Ms Gillian Mayer	REC Manager
Mr Neil McCaffery	Deputy Regional Manager
